# Effects of the first COVID-19 lockdown on quality and safety in mental healthcare transitions in England

**DOI:** 10.1192/bjo.2021.996

**Published:** 2021-08-31

**Authors:** Natasha Tyler, Gavin Daker-White, Andrew Grundy, Leah Quinlivan, Chris Armitage, Stephen Campbell, Maria Panagioti

**Affiliations:** NIHR Greater Manchester Patient Safety Translational Research Centre, University of Manchester, UK; NIHR Greater Manchester Patient Safety Translational Research Centre, University of Manchester, UK; School of Health Science, University of Nottingham, UK; NIHR Greater Manchester Patient Safety Translational Research Centre, University of Manchester, UK; and Centre for Mental Health and Safety, University of Manchester, UK; NIHR Greater Manchester Patient Safety Translational Research Centre, University of Manchester, UK; Manchester Centre for Health Psychology, University of Manchester, UK; and Manchester Academic Health Science Centre, Manchester University Foundation Trust, UK; NIHR Greater Manchester Patient Safety Translational Research Centre, University of Manchester, UK; NIHR Greater Manchester Patient Safety Translational Research Centre, University of Manchester, UK

**Keywords:** Inpatient treatment, qualitative research, COVID-19, care transitions, quality and safety

## Abstract

**Background:**

The COVID-19 pandemic forced the rapid implementation of changes to practice in mental health services, in particular transitions of care. Care transitions pose a particular threat to patient safety.

**Aims:**

This study aimed to understand the perspectives of different stakeholders about the impact of temporary changes in practice and policy of mental health transitions as a result of coronavirus disease 2019 (COVID-19) on perceived healthcare quality and safety.

**Method:**

Thirty-four participants were interviewed about quality and safety in mental health transitions during May and June 2020 (the end of the first UK national lockdown). Semi-structured remote interviews were conducted to generate in-depth information pertaining to various stakeholders (patients, carers, healthcare professionals and key informants). Results were analysed thematically.

**Results:**

The qualitative data highlighted six overarching themes in relation to practice changes: (a) technology-enabled communication; (b) discharge planning and readiness; (c) community support and follow-up; (d) admissions; (e) adapting to new policy and guidelines; (f) health worker safety and well-being. The COVID-19 pandemic exacerbated some quality and safety concerns such as tensions between teams, reduced support in the community and increased threshold for admissions. Also, several improvement interventions previously recommended in the literature, were implemented locally.

**Discussion:**

The practice of mental health transitions has transformed during the COVID-19 pandemic, affecting quality and safety. National policies concerning mental health transitions should concentrate on converting the mostly local and temporary positive changes into sustainable service quality improvements and applying systematic corrective policies to prevent exacerbations of previous quality and safety concerns.

## Background

The transition of care is widely regarded as a vulnerable ‘pinch-point’ in the patient journey.^1^ Mental healthcare transitions in particular pose a major threat to patient safety^2–4^ with ineffective discharge from acute mental health services associated with the increased likelihood of adverse outcomes, such as suicide and self-harm.^4,5^ Even where there is good integration, the pressure on all services can still make transitions problematic, i.e. lengthy waits for support in the community.^6^ Increased demand for acute mental health services creates pressure to discharge, but limited integration with community health and social care settings often makes timely and safe discharge challenging.^3,7,8^

The coronavirus disease 2019 (COVID-19) pandemic forced the rapid implementation of several practice changes in mental health services in *ad hoc* ways^9^ and amendments in the operationalisation of the Mental Health Act.^10^ NHS England and NHS Improvement guidance at the beginning of the pandemic suggested ‘providers may need to make difficult decisions in the context of reduced capacity and increasing demand. These decisions will need to balance clinical need (both mental and physical), patient safety and risk’.^11,12^ These rapid changes may drastically have affected the quality and safety at mental healthcare transitions. For example, expedited discharges from mental health services to social/community care were implemented in the UK and other European countries because early projections showed that in-patient bed capacity could be overwhelmed because of COVID-19^9,10,13–15^ Similarly, in the UK a 30% reduction in primary care consultations, and significant drops in referrals to mental health services were observed in the first wave of COVID-19.^9,14^ Moreover, in April 2020 new diagnosis of depression and anxiety was considerably reduced and referral to mental health services was less than a quarter of the expected rate for the time of year.^16^

The literature also suggests that the immediate stress and psychological impact of COVID-19 was worst for those with psychiatric illness than those without.^17^ Thus, opportunities to monitor psychosocial needs and support patients directly in clinical practice, community and primary care were greatly curtailed by large-scale home confinement.^18^ The use of digital technology to enable patient consultations and facilitate interprofessional communication were available prior to the pandemic but were used more extensively during the pandemic with potential benefits and drawbacks in terms of equity, quality and safety.^19^ However, the full range of changes that may have transformed mental health transitions during COVID-19 and the impact of these changes on the quality and safety of mental healthcare transition have not been investigated.

## Aims

This study aimed to understand the perspectives of different stakeholders’ (patients, family/informal carers, healthcare professionals, key informants) about temporary changes in practice during mental health transitions as a result of the first COVID-19 lockdown, and the impact of these changes on perceived healthcare quality and safety. This understanding could help improve quality and safety of mental healthcare transitions during and in the aftermath of the COVID-19 pandemic and contribute to the effective resolution of future healthcare crises.

## Method

### Design

Semi-structured interviews were conducted to generate in-depth information pertaining to participants’ experiences and viewpoints of mental healthcare transitions during the end of the first UK national lockdown in the COVID-19 pandemic. The study primarily sought to understand these perspectives to inform the development of a best practice intervention for improving the quality and safety of mental health transitions. Verbal informed consent was obtained from all participants. The study has been approved by the Health Research Authority (HRA) and Health and Care Research Wales (20/NW/0228). This study was performed in accordance with the Declaration of Helsinki. All adult participants provided verbal consent to participate in this study and this was approved by HRA and was because the COVID-19 pandemic meant face-to-face interaction was not possible. The verbal consent process was recorded/documented by audio recording and transcription at the beginning of each interview.

### Recruitment, sampling and participants

A purposive sampling strategy was used to identify groups and individual stakeholders with experience and knowledge of discharge from acute mental health services (*n* = 14; pharmacists, nurses, ward managers, occupational therapists, clinical psychologists, healthcare assistants, psychiatrists); patients (*n* = 6), carers (*n* = 7) and key informants (*n* = 7) (policymakers, academics, third sector workers, service managers). In total 34 participants were interviewed, Supplementary file 1 available at https://doi.org/10.1192/bjo.2021.996 describes participant sociodemographic information.

Participants self-identified from recruitment adverts posted on social media, sent by email or a personal approach from a member of the research team's professional network. Participants completed a screening pro-forma ahead of the interviews to ensure they had sufficient knowledge of mental health transitions with a focus on in-patient/acute services. Participants were reassured that the raw data would be anonymised upon transcription.

### Data collection

Interviews were conducted in May and June 2020 using online videoconferencing software or via telephone and at times convenient for the participants. Interviews lasted for approximately 45–60 min and were audio-recorded and transcribed verbatim. Two members of the research team conducted all of the interviews (N.T., G.D.-W.).

A semi-structured topic guide (see Supplementary file 2) was used to steer the discussion within the interview. The content was informed by existing interventions (i.e. SAFER,^20^ discharge teams, patient written discharge plans), previous literature and the experience of members of the research team of working in mental health services.^3,8,20,21^ The semi-structured approach enabled us to undertake a ‘conversation with purpose’ with parallel flexibility to discussing issues that may be particularly relevant to each participant.^22^

The discussion around COVID-19 arose organically using the topic guide that was approved before the pandemic. As interviewees were interviewed in the very early stages of the pandemic, it would be impossible to ignore the impact of COVID-19 and the semi-structured approach allowed us to prompt at each question for pre- and post-COVID-19 changes.

### Analysis

We collected basic, descriptive data around participant demographics (i.e. age, gender, stakeholder group, professional title). The qualitative data were transcribed verbatim and analysed thematically using conventional qualitative techniques proposed by Ritchie & Spencer.^23^ First, any data concerning COVID-19 was extracted into a separate database. This was an inductive approach that involved coding the individual participant responses and then grouping these together as ‘meaning units’. These grouped units were then assigned consolidated codes and the similarities and differences between them were compared. A further consolidation and consideration process led to the development of overarching themes to explain the data in terms of healthcare quality and safety. We did this by presenting any key themes that related to quality and safety risks/threats/debates in mental healthcare transitions or patient safety literature that were perceived by respondents to have changed because of COVID-19. The development of themes and analysis was led by N.T. and then discussed virtually within the wider research team for verification purposes. The team comprised academics and peer researchers that specialised in mental health safety, care transition safety or both.

### Patient and public involvement

Two patient and public involvement members were involved throughout the design and conduct of this research including appraising and suggesting changes to the topic guides. An expert by lived experience (peer researcher) contributed to the design, conduct and analysis, and manuscript preparation. Contributors were remunerated according to INVOLVE guidelines.^24^

## Results

Six overarching themes in relation to COVID-19 practice changes and associated impact on the quality and safety of mental health transitions were identified. The Appendix summarises the themes and subthemes, Supplementary file 3 includes more detailed information with exemplar quotes.

### Technology-enabled communication

#### Multiprofessional interagency teams

Most healthcare professionals felt that the initial wave of COVID-19 had been a catalyst for an immediate switch to virtual technologies for interactions between interagency teams. The widespread use of virtual technology was largely considered beneficial because:

- of reduced travel time;
- of strengthened interpersonal relationships;
- of greater attendance by professionals located outside of in-patient services; and
- it was easier to co-ordinate.

For example, professionals described how increased availability of healthcare professionals (because of reduced diaries as face-to-face contact was no longer necessary) facilitated interagency working.

‘Things are actually better under lockdown in a weird kind of way, because you're not having to arrange a time for a community person or for carers to actually physically be there. So there is more chance of a joint conversation between the in-patient team and the community team and the service user and their family.’ (Inpatient clinical psychologist, P19)

However, drawbacks were also reported, for example, the ease of communicating with professionals in the next office or corridor was removed by increased home working and reliance on virtual communication. Engagement was sometimes less than had it been face-to-face and missing calls/technology failures were common.

Tensions were described between in-patient and community teams associated with differing levels of exposure and risk expected from each service:

‘I'm here and I'm seeing people face to face. And then other community teams aren't seeing service users at all … It's really aggravated tension between different teams.’(Inpatient psychiatrist, P34)

Hence, the COVID-19 crisis sometimes highlighted and aggravated existing tensions between teams.

#### Communication with and involvement of carers

Professionals felt that technology facilitated carer involvement in discharge planning, but carers and patients focused on the negative effect of reduced/cancelled ward visits, although they recognised the practical usefulness of technology:

‘people can't be there in person to plan care and discharge. And you could do it on the phone but it's not the same.’ (Patient, G2)

Carers also describe being left out of communication regarding discharge planning and left alone to cope with family members who were not necessarily ready for discharge. Some carers described the initial perceived rapid discharge of patients at the beginning of the pandemic as distressing.

‘It was all so sudden because we literally had a phone call on a late Tuesday afternoon saying that he was being discharged and asking us could we go and collect him … he was handed over to us in a carpark, I asked, well, I asked specifically is this due to COVID and they said, oh no. Literally it was. It was all quite surreal. And I can laugh about it now. The psychiatrist saw us from his office and he came out to say good luck and goodbye…it was all a bit odd.’ (Carer, G4)

Moreover, adherence to social distancing guidelines that affect the physical act of ‘leaving hospital’ were considered a triumph for management, but the social distancing practices may have exacerbated carers’ and patients’ perceived lack of communication regarding discharge.

‘We've maintained escorting our service users off the ward and adhering to all the government guidelines for social distancing.’ (Ward manager, G1)

#### Communication between staff and patients

The communication between professionals and patients throughout the care pathway and transitions of care changed considerably with increased reliance on virtual technology. Benefits reported by professionals and patients included increased attendance at follow-up meetings because of reduced travel or social obligations:

‘it's really easy, ’cause I just sit on the sofa, and I don't get any pressure from anybody.’ (Patient, P26)

Professionals and patients also described drawbacks such as difficulties of capturing non-verbal communication and assessing physical environmental cues, and also mechanical discussion of sensitive issues:

‘when you are sat in front of somebody you can gauge a lot better in how they are.’ (Patient, P31)

Difficulties in effectively communicating with certain groups (i.e. vulnerable adults, homeless, those with technology paranoia, older adults) were also highlighted:

‘It really doesn't work for people who have paranoia about tech.’ (Patient, G2)

Many services assumed that they can just ‘go online’ but this was not ideal for many patients.

### Discharge planning and readiness

The importance of adequate discharge planning during COVID-19 was highlighted by all participants otherwise people would miss out on care along the pathway because of limited community resources:

‘If wards were being closed very suddenly I just can't see that having been done, all those links with housing and social services and benefits and then stepping care … So it was worrying me that those problems are there generally for inpatient discharge, but if we're going to rush discharges because of an emergency situation that was going to make everything a hundred times worse.’ (Patient, G2)

The COVID-19 pandemic forced better planning of the social elements of discharge according to health professionals, which was viewed as beneficial. One social factor in particular highlighted was having carers involved in discharge planning and post-discharge care.

‘we've had to think about now with COVID, if you're discharging someone who's clinically vulnerable to COVID it's thinking about can they go to a supermarket and buy food, and if not making sure that some arrangement is made beforehand. So sometimes it's nothing to do with mental health, it's just social factors.’ (Head of nursing, P33)

Participants concurred that the emergency nature of the pandemic sped up discharge processes because the main causes of delayed discharge pre-pandemic, were interagency disputes over social issues (rather than health issues) and limited resources and overreliance on national guidance.

For example, professionals described that having access to more funding and fewer bureaucracies during the pandemic have made the discharge planning process easier:

‘by bypassing the normal, hugely prolonged, totally unnecessary funding procedures, off the back of COVID money, so it just proves that they can do it without the need to create thousands of pieces of paperwork.’ (Nurse, P2)

Similarly, professionals felt that the risk of COVID-19 outweighed other risks surrounding inappropriate discharge, meaning discharges that would have ordinarily been delayed, happened, as staff were less reluctant to discharge. Furthermore, guidance surrounding discharge quality and safety is usually issued on a national level, policy advisors felt that the non-existence of national guidance forced teams to work together to implement local solutions:

‘it was a real impetus for local authorities and the trusts to work together effectively … to sort these issues out and there's no national, you know, accountability for it.’ (Third-sector policy advisor, G7)

However, some participants reported slower processes because of the removal of components that facilitate speedy discharge, such as leave and family visits, indicating that positive effects were localised or were not applicable to all patient groups. Furthermore, many interviewees, particularly patients, highlighted concerns over the safety of rapid discharges:

‘I'd heard a few professionals and academics saying that wards had been cleared very abruptly because of COVID and they spoke of there being no ill effects, and I felt like, okay, if you're measuring it against no suicides, no serious untoward incidents, no criminal activity maybe it looks okay, but is anybody talking to those service users about the impact on their lives of being summarily discharged into the community because either they were well enough to be discharged anyway or they weren't and they shouldn't have been discharged.’ (Patient, G2)

### Community support and follow-up post-discharge

A key safety concern expressed by healthcare professionals, was that individuals who were discharged early were left to cope in the community following the closure of many community support services or movement towards virtual meetings. Similarly, patients and carers experienced significant changes in terms of frequency and composition of community support.

‘So the service user group … used to happen twice a week, that's all gone and there's nothing that's been put in place to help people be able to talk to each other. So you've got a whole load of service users who have basically been booted out of the hospital early … who have just been abandoned.’ (Carer, G3)

All groups of participants expressed concerns about the effect of the closure of post-discharge community services in terms of increased psychological distress or increased admissions and pressure on acute services:

‘the community and mental health team … they had to cancel obviously and then re-arrange, which didn't help with my anxiety.’ (Patient, P18)

‘An influx of mental health [presentations] because people aren't able to engage with the community teams … The follow up from discharge has just been patchy, very patchy.’ (Psychiatrist, P34)

### Admissions

Participants described reduced rates of admissions as a remarkable change during the first COVID-19 UK lockdown**.** Many participants described an increased threshold for in-patient admission because of limited bed numbers, ward closures, increased need for in-patient care as community services closed and fears of spreading the virus. The threshold was considered even higher for older adults:

‘it's easier for a camel to pass through the eye of a needle than to get older adults admitted.’ (Psychiatrist, P34)

Patients and carers described how they 'worked harder' to avoid hospital admissions due to a fear of catching the virusin an in-patient setting or the effect of new procedures (e.g. personal protective equipment) on mental health. There was also a fear that new procedures would make the environment less safe.

‘Yeah, for me I've definitely been working harder on my own mental stability to make sure that I don't end up being admitted, because I think that would be an absolute nightmare in the current climate, particularly if people are all geared up in PPE [personal protective equipment].’ (Patient, P35)

However, a major safety concern related to reduced admissions, was that people continued to be unwell but still chose not to seek help:

‘we had a big drop in the number of service users being admitted for a while, so you wonder where they are because they're still unwell.’ (Pharmacy advisor, P27)

### Integrating new policy and guidelines

The crisis resulted in many changes in policy, legislation, guidelines and practice. Some examples are the introduction of PPE for staff, self-isolation and social distancing.^14^ These changes were considered difficult during transitions, particularly for increasing post-discharge feelings of loneliness among patients with paranoia and anxiety.

‘in some ways all your fears about being on your own in the community after discharge are actually reality now because you have to self-isolate and you have to be careful and not trust people and stay away from people.’ (Care Quality Commission inspector with lived experience, P28)

Professionals described having to educate patients who were admitted before the pandemic upon discharge of new ways of living in the community such as social distancing:

‘We've implemented lots of things to help support our service users during COVID and then, you know, working towards going home. Because I had service users that were in before the lockdown and so for them they had no idea about social distancing and those sorts of things.’ (Nurse, G5)

Participants also described how the pandemic affected the care pathway, whereby many hospitals in an effort to create socially distanced wards, introduced transition wards or corridors whereby patients awaited test results before discharge or upon admission:

‘they're all admitted to one corridor, an isolation corridor, and they stay in there…until such time as their COVID test result is back.’ (Nurse, P2)

### Health worker safety and well-being

Throughout the interviews two key threats to health worker safety and well-being were reported. The first safety issue for health workers was the lack of PPE and the resulting fear of spreading the virus to their families and patients/carers:

‘it's probably had a hugely exhausting impact for people in inpatient settings. I think all services across the NHS [National Health Service] have had to think about how to try and continue to do their routine business and adapt for working during COVID, and thinking about this next phase of COVID.’ (Clinical psychologist, P36)

The second issue was the damaging effect of the multiple technology-enabled meetings:

‘people are in back to back meetings for hours at a time, which isn't ideal … I don't think that's fantastic for the wellbeing of staff.’ (Clinical psychologist, P19)

These multiple meetings increased the stress levels of health workers and sometimes resulted in communication tensions/failures, which in turn were perceived to affect the quality and safety of care transitions:

‘Yeah, I think probably communication with the wards has not been as good. I think ward staff are really, really stressed, so CPAs [Care Programme Approach] haven't always been happening when they're supposed to be.’ (Occupational therapist, P29)

## Discussion

### Summary of findings

Our findings suggest that the COVID-19 pandemic (in particular, the first UK lockdown) has enforced major changes in mental healthcare transition practices and policies that have exacerbated existing patient care quality and safety concerns or have highlighted new quality and safety concerns. Technology-enabled meetings often resulted in tensions between staff members, might have limited potential to build/maintain rapport between health professionals and patients, and gather meaningful input by carers concerning discharge decisions, and are not well suited for patients with specific conditions such as paranoia or people with low e-literacy.

The quality of discharge and admission planning was perceived to be suboptimal in response to the national urge to free-up hospital beds, resulting in discharging patients from hospitals who were not ready to cope in the community or not admitting patients who needed in-patient care. Patients and their carers described hopeless moments of discharge where they have been wished ‘goodbye and good luck’ by their consultant without being able to access help in the community. The parallel closure of most community support services meant that patients had minimal opportunities for accessing care via alternative routes worsening their feelings of helplessness and loneliness.

Despite this, several of these changes could be resolutions to pre-existing safety threats. For example, technology-enabled meetings have enabled interdisciplinary teams and agencies to jointly discuss patient discharge. Collaborative working between multidisciplinary/multiagency teams to improve discharge planning was previously a core element of integrative care models, yet widespread implementation was previously considered unrealistic.^8^ Technology-enabled meetings also improved patients’ attendance for post-discharge follow-up meetings as practical barriers were eliminated (such as travelling complications, social phobia). The emphasis on social factors in deciding discharge readiness was perceived positively as social factors are often responsible for delayed discharges.^25^ Moreover, providing the resources and the responsibility to local health and care teams to create their own discharge planning policies has led the rapid implementation of interventions that reflected the need of their wards.

### Strengths and limitations

As interviews were conducted during the first English national lockdown, it provides real time accounts of the effect of the initial stages of COVID-19 pandemic on care transitions highlighting perspectives of multiple stakeholders. However, this study also has limitations. One key limitation of this study is that despite planning to recruit participants from three selected NHS sites, the emergence of the pandemic meant we recruited through social media and not directly through NHS sites, trusts were already under pressure and research was halted therefore participants self-selected in response to social media adverts. Additionally, although our study provides useful insights into stakeholder perspectives and experiences at a personal and local level, large-scale studies are needed to explore and confirm these findings nationally. This study benefits from presenting perspectives of a relatively large, stakeholder group that includes multidisciplinary professionals and highlights the voices of patients and carers. However, numbers in each stakeholder subgroup were relatively low; it is likely that a larger number of interviews with patients and informal carers especially could contribute richer data to the analysis. Furthermore, only 3 of the 34 participants were from ethnic minority groups.

Our data have been collected during the first wave of the pandemic and mostly concern temporary changes rather than permanent changes to services. We strongly recommend a mixed-method follow-up study and/or an audit to see service change implementation in terms of discharge rates/speed, telecommunication, admissions and community follow-up. Such study would be very useful in assessing (a) the effects of these changes and (b) the permanency of the changes to service. Despite this, most of the safety concerns identified in this study appear to be valid throughout the pandemic.^2^ For example, a recent study highlighted that the consequences of reduced clinical contact on self-harm are still unknown.^26^ Moreover, our recent consultations with patient and public involvement members have showed that concerns around rapid discharges, accessing follow-up/community care, accessing in-patient services and use of technology were still current and valid problems from the perspective of patients and carers, 12 months after the pandemic began.

### Clinical and research implications

The headline implication of this study is that critically dangerous safety failures in the care pathway (lack of community support at post-discharge, low engagement of carers and exacerbation of health inequalities for the most vulnerable patients such as those with low e-literacy or paranoia) were considered by patients and carers to be even more likely to occur during the pandemic. In contrast, the improved interprofessional/agency communication, the reduction in delayed discharges and the integration of social factors on discharge planning have been remarkable achievements.

The immediate implementation of new communication processes to enable mental healthcare transitions is unprecedented in the UK and greater use of technologies and/or remote working, are likely to permanently change the way mental health services work.^27^ The use of digital technologies for treatment is also emerging in the literature, for example cognitive–behavioural therapy.^28^ The Nuffield Trust published guidance about the uptake of remote technologies, highlighting how important innovative local approaches to technology-enabled communication has been, but also highlighting the risks associated with rapid uptake and limited guidance from central bodies.^29^ A major caveat is that technology-enabled communication is seen as an oversimplified, one-size-fits-all solution.^30^ The readiness of services (i.e. reliable internet connection, hardware and technical support) and readiness of patients (e-health literacy, paranoia, potential harm caused by missed appointments at post-discharge)^27,30^ in using technology needs to be thoroughly assessed and addressed.

Multiagency, digitally enabled meetings between professionals offers a structured, optimal communication method between services that could have multiple quality and safety benefits and it is likely to become a permanent practice. Patients and professionals agreed in a pre-pandemic study from 2019 that joint meetings of multiprofessional and interagency teams is a potential solution to the communication issues in regards to mental health discharge^8^ but these had only been tested at local scale.^8,31–33^ However, team climate improvements are needed to help establish multiprofessional and multiagency teams that function effectively. Uncontrolled power dynamics and conflicting perspectives within such teams could cause stress to staff members and reduce the meaningful contribution of informal carers. Moreover, technology-enabled communication can reduce safety threats for those people for whom travelling poses complications or people with social anxiety/phobias but it might increase health inequalities (for example low e-literacy or paranoia.)

Discharge planning from mental health services to the community became speedy and efficient and could potentially drive long-term practice changes. Key enablers are the increased consideration of social factors in assessing the discharge readiness of patients, greater availability of funding/resources and the implementation of local policies/interventions to which staff had better ownership (staff engagement or motivation to change practice has been described as a key barrier to implementing new interventions in the past).^34^

Key caveats are undermining the safety implications of discharging patients too early, not involving carers in discharge planning and lack of community services. The closure of community support services because of the first COVID-19 lockdown across the English national health and social systems could be associated with fatal consequences, before the pandemic (in 2018) 13% of deaths by suicide in the UK are in people recently discharged from mental health services,^35^ however, since our data was collected, recent research suggests that there was not an increase in suicide rates in the months following the first lockdown.^36^ Recent evidence highlights the need for accessible healthcare services,^26^ remote assessment and care pathways for vulnerable patient groups, and training staff members in the new ways of working.^37^

An international group of clinicians, mental health experts, and users of mental health worked together on a recent paper to highlight how mental healthcare should change as a consequence of the COVID-19 pandemic.^38^ The group proposed that sustainable adaptations of systems and mental healthcare should be developed collaboratively by experts, clinicians and patients. They also described how they should be designed to mitigate disparities in healthcare provision and highlighted the importance of continuous assessment of health and service-use outcomes for deciding which practices should be continued and developed or discontinued. Overall, enablers and caveats should feed into future policy and practice including the implementation of existing NHS Improvement solutions aiming to systematically assess discharge readiness, improve discharge planning and enable early patient discharge where possible. The Department of Health and Social Care recently published the COVID-19 Mental Health and Recovery Action Plan; which focuses on and includes funding for ‘Continued commitment to the expansion and transformation of mental health services’, therefore it is hoped that Government funding and policy initiatives to improve services result in meaningful changes to services for patients going forward.^39^

From the earliest days of the pandemic, there was a concern that suicide and self-harm would increase because of reduced access to services during the first lockdown,^36^ however, initial data does not show this. Current literature suggests there was no significant rise in suicide figures post-lockdown.^36^ Primary care records indicate that the incidence of self-harm in the UK was 37% lower than expected in April 2020, but by September 2020 it was similar to expected levels.^16^ The consequences of reduced clinical contact on self-harm and suicide beyond September 2020 are still unknown.^16,36^ On 29 April 2020, NHS England guidance was released that focused on seven high priority areas, one of these was to proactively contact and support existing patients known to mental health services,^11^ however, patients in our study did not report the effects of this guidance in May–July 2020. Future research should consider how to ensure the effects of rapidly developed national guidelines are translated into meaningful practice, as patients did not report proactive contact that was proposed in the guidleines.

In conclusion, our study found that the COVID-19 pandemic exacerbated some quality and safety concerns in mental healthcare transitions such as tensions between teams, reduced support in the community and increased the threshold for admissions. However, the emergency situation fast-tracked localised implementation of evidence-based interventions for increasing the quality and safety of mental health transitions such as streamlining discharge readiness, increased use of technology-enabled multiagency communications and greater consideration of social factors in discharge planning. The core challenges for future policy and practice in relation to mental health transitions are to (a) implement corrective policies to prevent adverse patient outcomes caused by the increase of some existing quality and safety concerns and (b) transform the mostly local and temporary positive solutions that emerged in the COVID-19 pandemic into sustainable service quality improvements.

## Data Availability

The data that support the findings of this study are available from the corresponding author (N.T.) upon reasonable request.

## Supplementary material

For supplementary material accompanying this paper visit http://dx.doi.org/10.1192/bjo.2021.996. click here to view supplementary material

## Appendix

A summary of the themes and subthemes, divided into changes generally considered beneficial by stakeholders and quality and safety concerns

**Table:**

Theme	Subthemes	
	Beneficial changes and opportunities for sustained improvement	Quality and safety concerns and opportunities for preventative actions
**1 Technology-enabled communication**	*Functioning of multiprofessional interagency teams*	*Functioning of multiprofessional interagency teams*
	Use of technology has improved communication between teams (attendance, removing physical travel and busy diaries) Pandemic ‘togetherness’ and video technology building relationships and rapport between teams	Increased tensions between community and in-patient professionals about differences in risks taken Hard to get interagency teams to attend online meetings as seen as less important (technology enables people to not fully engage in discussions)
	*Communication with /involvement of carers*	*Communication with /involvement of carers*
	Involvement of carers in telecommunication meetings	Carers/family not able to visit wards for discharge planning Carers left out of communication at the beginning of crisis Staff perceptions of socially distanced handover as following guidelines
	*Communication between staff and patients*	*Communication between staff and patients*
	Improved attendance in follow-up meetings (technology removes travel complications and social anxiety for some)	Difficult to develop rapport, fully assess social cues and personal situations (i.e. how the house looks) with telecom Telemedicine not ideal for older adults and vulnerable groups Effect for people with paranoia and voices of zoom Removing group sessions and allowing patients to engage from sofa, can reduce anxiety and be beneficial for some (community follow-up)
**2 Discharge planning and readiness**	More consideration of social elements post-discharge including education about COVID-safe behaviours, checking accommodation and family circumstance Carers more important than ever as less community services to discharge to Emergency situation has removed some of financial reasons that cause delayed discharge Emergency situation removing staff's reluctance to discharge due to risk Problem solving on a local level without overreliance on national guidance sped up transitional processes	Discharge planning in an emergency situation resulting in more opportunities for people to fall through the cracks Concerns about rapid mass discharges Some wards reported discharges slowed down because of reduced leave and visitation or isolation before discharge
**3 Community support and follow-up post-discharge**		Reduced support in community (during transitions and in general) Concern for patients asked to cope in the community that might usually be receiving acute care
**4 Admissions**	Numbers of new admissions have been reduced improving in-patient bed capacity	Patients feeling scared to be admitted and avoiding hospital Initial reduction in admissions Increased thresholds for admission because of ward closures, reduced beds, fears of spreading virus and increased need because of lack of community care Exacerbated difficulties with admitting older adults
**5 Adapting to new policy and guidelines**	Introduction of new services/wards (‘transition’ wards and mental health accident and emergency) Educating patients about COVID-19 guidelines (social distancing etc.) after discharge	Effect of personal protective equipment (PPE), social distancing and isolation on mental health symptoms and loneliness during transitions Reduction in beds because of creating a socially distanced environment
**6 Health worker safety and well-being**		Stressed ward staff affecting communication with other agencies Ill effect of back-to-back telecommunication meetings Stress of adapting services and dealing with patient anxieties Stress of lack of PPE and spreading virus to families
