# Supplementary material for: Effects of the first COVID-19 lockdown on quality and safety in mental healthcare transitions in England
Source: BJPsych Open. 2021 Aug 31;7(5):e156. doi: 10.1192/bjo.2021.996 (PMC8410739; doi:10.1192/bjo.2021.996)
Supplement: Supplementary file 1 [file bjosup.zip › S2056472421009960sup001.docx]

Existing latent factors associated with threats to patient safety

| Theme | Subtheme | Example Quotes |
| --- | --- | --- |
| Communication between multi-professional inter-agency teams | Use of technology has improved communication between teams (attendance- removing physical travel and busy diaries)  Pandemic ‘togetherness’ and video technology building relationships and rapport between teams  Increased tensions between community and acute about differences in risks taken  Hard to get inter-agency teams to all attend online as seen as less important (technology enables people to not fully engage in discussions) | ‘I think things are actually better under lockdown in a weird kind of way, because you’re not having to arrange a time for a community person or for carers to actually physically be there. So there is more chance of a joint conversation between the inpatient team and the community team and the patient and their family’- inpatient Clinical Psychologist  ‘Yeah, that’s been a real positive. So we’ve always had a problem of getting community teams involved enough when someone’s in hospital, just because of the fact that CPNs book their diaries so far in advance that they can’t be that responsive, particularly at the beginning of an admission. And then the other thing is the time that it takes, because they’ll turn up to ward round, ward round inevitably is running late, so they’re hanging around half an hour before they even go in, then their ward round might take longer than it should have done’ – Head of nursing P33  I suppose on the Covid I mean it is really positive that that trust or authority was seeing things improve and it's really hard to know it in some ways because there has been this amazing drive and imperative to work together. – Third sector policy advisor G6  ‘I think it builds the relationship between the professionals because we're all, more often than not, in the same boat, you know, as we are when we're working from home, we've all got kids running around, we've all got…we're all trying to, you know, have meetings with our children here, and have lunch. I think that that does kind of understated wonders for the relationship between a community team and a hospital team.- OT  ‘I’m here and I’m seeing people face to face. And then other community teams aren’t seeing patients at all… It’s really aggravated tension between different teams.’- inpatient psychiatrist  But for some of the most complicated patients that we have – I don’t mean complicated in terms of their mental health, but in their social care needs and their physical care needs and occupational therapy and physical therapy and all sorts – you never get everybody at the same time now, and before I might have been able to have a professionals meeting. But it seems like if there’s a professionals meeting on Microsoft Teams it’s much easier to not engage in that you can sign in but you don’t have to have your face on there. I don’t know, it feels like there isn’t as much buy-in as there might have been face to face.- inpatient psychiatrist |
| Communication with /involvement of carers | Involvement of carers in telecommunication meetings  Carers/family not able to visit wards for discharge planning  Carers felt left out of communication at the beginning of crisis  ..But, socially distanced handover considered following guidelines perhaps without thinking about impact and perceptions on family | ‘because families now don’t have to take the afternoon off work. They could book 15 minutes out of their day at work to phone in and do the ward round. So that’s been a positive, and that’s something that we’ve said we’d quite like to keep doing’ – head of nursing P33  People are finding wards very difficult at the moment because of all the restrictions, and not being allowed visitors isn’t helpful because then relatives or carers can’t take part in ward rounds for discharge planning either, and it’s tremendously hard for the patients not to be able to have… You look forward all day to your visitor coming, and if that’s not going to happen that’s quite soul destroying. But it means people can’t be there in person to plan care and discharge. And you could do it on the phone but it’s not the same, and you could do it via Zoom, but it’s not - SU  ‘Well, because it was all so sudden because we literally had a phone call on a late Tuesday afternoon saying that he was being discharged and asking us could we go and collect him. I said we can't get there 'til tomorrow. And when they met us in the carpark, he was handed over to us in a carpark, I asked…well, I asked specifically is this due to COVID and they said, oh no… Literally it was. It was all quite surreal. And I can laugh about it now. The psychiatrist that had originally sectioned him but wasn't the psychiatrist that had discharged him, saw us from his office and he came out to say good luck and goodbye, and was a bit sort of…he seemed a bit embarrassed actually, it was all a bit odd,’ – carer  ‘We’ve maintained escorting our patients off the ward and adhering to all the government guidelines for social distancing.’ – ward manager |
| Adequacy and Frequency of community support and follow up post-discharge | Very little support in community  Concern for patients asked to cope in the community that might usually be under acute care | ‘ So the service user group, the [name] group, that used to happen twice a week, that’s all gone and there’s nothing that’s been put in place to help people be able to talk to each other. So you’ve got a whole load of service users who have basically been booted out of the hospital, especially with COVID because they closed so many wards, who have just been abandoned, as far as I can see.’ Carer- G3  An influx of mental health because people aren’t able to engage with the community teams… The follow up from discharge has just been patchy, very patchy, which I think has been really detrimental to patient care. - Psychiatrist P34  ‘It was just chaos for the mental health services, obviously. A lot more appointments, a lot more difficult meeting with the actual service that you were going to see. I know I myself had a meeting in town to go and see, I can’t remember their name, but…it was the Community and Mental Health Team, that was it, but they had to cancel obviously and then re-arrange, which didn’t help with my anxiety, because I didn’t hear about the cancellation until about an hour before.’ – Service User P18  So the support that you get on discharge is less, and some services aren’t available, so things like the peer support workers and stuff that they do in the community, they’re just not doing anything at the minute. Because it’s not an essential service they can’t do it. So it’s very weird that there’s almost less in the community but we’ve discharged more people, and we’ve probably discharged them earlier than we would, and in theory it should be the other way round.- Head of Nursing- P33  I think we've had less patients, well, we've now gone back up again as expected, we knew there would be a peak but we had a big drop in the number of patients being admitted for a while, so you wonder where they are because they're still unwell- Pharmacy advisor – P27  So the number of people getting to crisis point because of the lack of support in community services and charities and face-to-face contacts has impacted how many people need the beds, which is inevitably going to impact how many people can have them, you know. – Occupational Therapist P29 |
| Delayed Discharge | Emergency situation has removed some of financial reasons that cause delayed discharge  Emergency situation removing staffs reluctance to discharge due to risk  Emergency situation resulted in problem solving on a local level to speed up processes, without national guidance  Some wards reported discharges slowed down due to reduced leave and visitation or isolation before discharge  SU concerns about rapid mass discharges | In a way it's actually…I mean it feels like more resources have become available because the barrier to discharge tends to be social but since more resources have become available it's just been a lot easier and we can just discharge a patient to a bed and breakfast, make sure that their care coordinator is aware and provides the patient with support in the community setting. – Psychiatrist P23  there’s this big Bed Bureau business, where people are supposed to be trying to find beds for people that need residential and nursing care quicker, by bypassing the normal, hugely prolonged, totally unnecessary funding procedures, off the back of Covid money, so it just proves that they can do it without the need to create thousands of pieces of paperwork…so I think that it probably proves that it’s probably not necessary, a lot of it….waiting weeks for this financial thing to be done, that financial thing to be done. It clearly doesn’t need to be done, does it, ‘cause they’ve done it literally, sometimes, within days. And I think they just speeded everything up, so it just proves that it can be done really.- Nurse P2  But [name] ward went down to something like five patients at the very beginning, from just over 20. And a lot of the staff were saying to me why could we discharge these people now when we couldn’t discharge them before, because from a mental health perspective nothing’s changed. I was saying, well, maybe this is practice that will continue. Because the biggest barrier I think to discharging people I think is actually the staff’s attitude and the staff’s reluctance to take risks and the fear of that if we had to be up in coroners court and that kind of thing. I think because COVID kind of almost shut that down and it was just you’ve got to do it, I think having done it and realised that we haven’t had more serious incidents in the community, we haven’t had an increase in suicides. We’ve had them, but we’ve had them in the way that we would normally have them.- Head of Nursing P33  Things have improved since coronavirus because that's been the need to clear beds or discharge people because of concerns it would spread around the ward and you'd have people at higher risk of catching it being an inpatient, that processes were speeded up and it was a real impetus for local authorities and the trusts to work together effectively. You know, using things like teams and Zoom for calls and just streamlining processes, holding more assessments, looking at assessment processes and really focusing on how things could be sped up and how to improve things. And they were actually quite positive, they were amazed. And I think that's partly it, was there isn't a national drive to…it seems to be down to local areas about whether they decide to…you know, to have plans in place to work effectively together to…you know, with NHS and local authorities to sort these issues out and there's no national, you know, accountability for it.- Third Sector Policy advisor- G7  ‘Because of movement visitors are not being allowed, it is not safe for the patients to go again to, into the community; so, I think it has, that has slowed down the process. It has actually slowed down the process. I find that a lot of the professionals have a little bit of difficulty reaching out to everybody, to contact’- nurse G8  So I think it's delayed…well, inevitably for some people it will mean that they're not in hospital for as long. And other people, it will mean they're there for a long time because people now have to go onto an acute assessment ward and then they have to stay there for 14 days. And then get moved again to another ward. So that's already really traumatic and you wouldn't want that, but it's so that they can kind of in some way quarantine people.- Occupational Therapist P29  ‘I’d heard a few professionals and academics saying that wards had been cleared very abruptly because of COVID and they spoke of there being no ill effects, and I felt like, okay, if you’re measuring it against no suicides, no serious untoward incidents, no criminal activity maybe it looks okay, but is anybody talking to those patients about the impact on their lives of being summarily discharged into the community because either they were well enough to be discharged anyway or they weren’t and they shouldn't have been discharged’ – SU G2 |
| Discharge Planning | More consideration of social elements post-discharge including covid behaviour education, checking accommodation and family circumstance.  Carers more important than ever as less community services to discharge to.  Discharge planning in an emergency situation resulting in more opportunities for people to fall through the cracks | ‘But that’s been a thing we’ve had to think about now with COVID, if you’re discharging someone who’s clinically vulnerable to COVID it’s thinking about can they go to a supermarket and buy food, and if not making sure that some arrangement is made beforehand. So sometimes it’s nothing to do with mental health, it’s just social factors’.- Head of nursing P33  Everybody who was in those wards including people like [name], who was the schizophrenic that we knew, the musician, he was basically just sent out. Apparently there was some amount of effort made in order to try and say, well, people should be going back to their families so people were sent back to their families where that could happen. People who didn’t have families to go to, people who were homeless, were basically moved out and [hospital] gave them a hotel room to stay in, requirements under the housing because they were homeless people, effectively, at that stage. Some of these people just don’t have anywhere else to go so we saw that happen.- Carer G3  ‘And it’s complicated to plan the transition from hospital back to community, and if wards were being closed very suddenly I just can’t see that having been done, all those links with housing and social services and benefits and then stepping care down maybe back to the crisis team and maybe step down from there to secondary care. I just can’t see how it could have all been planned properly if you’re just simply closing wards. My experience is that the more agencies are involved the more loopholes there are, the more cracks there are for people to fall down those cracks within what should be a kind of coherent system. So it was worrying me that those problems are there generally for inpatient discharge, but if we’re going to rush discharges because of an emergency situation that was going to make everything a hundred times worse.- SU- G2 |
| Admissions | SUs feeling scared to be admitted so avoiding hospital, leaving it longer so MH condition is worse than if arrived as usual or glad to be out of hospital  Initial reduction in admissions  Increased thresholds for admission due to ward closures, reduced beds, fears of spreading virus and increased need due to lack of community care  Exacerbated difficulties with admitting older adults | ‘Yeah, for me I've definitely been working harder on my own mental stability to make sure that I don't end up being admitted, because I think that would be an absolute nightmare in the current climate, particularly if people are all geared up in PPE. I guess depending on what that looks like, if I couldn't see someone's face, for example, and I could just see their eyes and you can't read how people are responding to you, so I think, yeah, I guess that's my greatest fear at the moment, is being admitted because of a deterioration’ – SU P35  ‘I think it’s separated the men from the boys a bit with people realising that they haven’t got an illness so they don’t need to go to the hospital.’ Service user- P18  ‘It’s a really bad time to be in hospital. Some trusts have said that if someone with suspected COVID, say they try to ligature and they go into cardiac arrest, normally the staff would perform CPR, but they’ve been told not to do that because of the risks of contracting COVID to staff. And then all the staff are in PPE so you don’t get the interpersonal relationship you would normally get particularly with nursing staff. So I’m really glad not to be in hospital at the moment.- SU G2  ‘There was a slight decrease in admissions for some, so there were much…there was certainly a big drop in people attending accident and emergency services, and I think that’s been nationally, and certainly people presenting in crisis to A and E departments and then requiring admission had decreased. So I think there’d been a bit less…there’d been slightly less admissions on the ward’- Clinical Psychologist P36  ‘I think a lot of patients stayed away for a while, and people who possibly would have come before stayed away because of COVID, and then got lots worse than they would have been if they’d come a bit earlier’ – Psychiatrist P34  ‘So the number of people getting to crisis point because of the lack of support in community services and charities and face-to-face contacts has impacted how many people need the beds, which is inevitably going to impact how many people can have them, you know.’ – Occupational Therapist  ‘As [name] was very, very sick and tried to cut his wrists [inaudible 33:12] came very close to dying at one point. That was about three weeks ago, four weeks ago or so. We tried to get him into a hospital at that point and were told that he could not go into hospital at that stage because he would only be admitted if he was sectioned and that he didn’t meet the criteria for being sectioned at that point.- Carer G3  ‘it’s just been harder to get people into hospital when they’re unwell…, I would say it’s easier for a camel to pass through the eye of a needle than to get older adults admitted’ – Psychiatrist P34 |
| Adequency and Frequency of communication between staff and patient | Improved attendance in follow up meetings (technology removes travel complications and social anxiety for some)  Difficult to develop rapport, fully assess social cues and personal situations (i.e. how the house looks) with telecom.) | ‘ So the outpatient psychiatry clinics have much improved, reported much improved attendance in April and May, for service users who might have been down as non-attenders or not being able to face getting on the bus or not being able to find a parking space, being able to be contacted.’ – clinical psychologist P36  ‘It’s so hard to pick up on people’s non-verbal communication over Zoom. And it is really awkward trying to assess people on a video.’- Psychiatrist P29  ‘Yeah, obviously when you are sat in front of somebody you can gauge a lot better in how they are and how they are dealing with things but we have got the technology to be able to have, you know, face to face calls so you can get a better idea.’ SU P31 |
| Adapting to new policy and guidelines | Introduction of new services/wards (‘transition’ wards and mental health A&Es)  Effect of PPE, social distancing and isolation on mental health symptoms during transitions  Reduction in beds due to creating a socially distanced environment  Educating patients about Covid guidelines (social distancing etc.) after discharge | ‘Yeah, I mean we’ve got quite an elaborate system on our acute ward, that we’ve set up, that everyone who gets admitted is, the number of beds is being reduced quite a lot during the Covid thing, and they’re all admitted to one corridor, an isolation corridor, and they stay in there, in their bedrooms, they have to consent to this, or even if they don’t want to consent, until such time as their Covid test result is back’.- nurse P2  And because of Covid…well, we were doing it before Covid, but we really pushed it, we're now almost having like mental health A&E remits on our acute sites, so encouraging rather than people to go to A&E they…they call them urgent care lounges where they can stay up to 24 hours, sometimes they stay longer but in theory it's 24 hours, where they can be assessed, and it's almost like an A&E for mental health, and I think that's going to be well established. – Pharmacy advisor – P27  ‘The paranoia is real so it’s completely bizarre and in some ways all your fears about being on your own in the community after discharge are actually reality now because you have to self-isolate and you have to be careful and not trust people and stay away from people and all those aspects of it. It’s like we’re living in a dystopia. ‘ – CQC inspector with lived experience P28  so it’s the most poorly people being asked to self-isolate, which you’re having an acute mental health crisis enough to warrant admission at this time, your chances of having capacity and actually being able to follow it through are quite limited. And then obviously if you are COVID positive then it gets even more restrictive. So, yeah, it’s challenging time- head of nursing P33  ‘So we’ve reduced our beds so that we’ve got two metres between every bed, and we created shielding wards and admission wards, and we’ve been quite strict on everything.’ – Head of nursing P33  ‘And very recently in fact we've implemented lots of things to help support our patients during COVID and then, you know, working towards going home. Because I had patients that were in before the lockdown and so for them they had no idea about social distancing and those sorts of things’ – Nurse G5 |
| Staff wellbeing | Stressed ward staff affecting communication with other agencies  Ill effect of back to back telecommunication meetings  Stress of trying to adapt services and deal with patient anxieties  Stress of lack of PPE and spreading virus to families | ‘ Yeah, I think probably communication with the wards has not been as good. I think ward staff are really, really stressed, so CPAs haven't always been happening when they're supposed to be.’ – Occupational Therapist P29  Unfortunately it does mean people are in back to back meetings for hours at a time, which isn’t ideal… I don’t think that’s fantastic for the wellbeing of staff,- Clinical psychologist P19  So certainly within adult services, I think in older adult services they were very concerned about the risk of Covid to…because with older people being so much more vulnerable, and then thinking then how to manage that with suspected cases and to…whether to move to a system of dedicated wards or trying to manage cases on existing wards, say to have a Covid ward or a non-Covid ward…So I think probably the…it’s probably had a hugely exhausting impact for people in inpatient settings. I think all services across the NHS have had to think about how to try and continue to do their routine business and adapt for working during Covid, and thinking about this next phase of Covid as things open up a bit more, how to offer services in a similar or different ways, and colleagues and inpatient settings have been very much on the frontline of that as well, and having to deal with the risk daily and the stresses of it and some service users’ wider anxieties about it as well. – Clinical Psychologist P36  There was delays with the PPE which was horrendous, with people several weeks after the start struggling to have the appropriate PPE, and then potentially placing themselves and others and family members at risk.- Clinical Psychologist P36 |
