# Supplementary material for: Effects of the first COVID-19 lockdown on quality and safety in mental healthcare transitions in England
Source: BJPsych Open. 2021 Aug 31;7(5):e156. doi: 10.1192/bjo.2021.996 (PMC8410739; doi:10.1192/bjo.2021.996)
Supplement: Supplementary file 1 [file bjosup.zip › S2056472421009960sup002.docx]

**Safer Mental Health Discharge: Stakeholder Interviews and Consensus Study**

**Topic Guide**

**Healthcare Professionals**

**Background**

You are a group of healthcare professionals working in mental health services and are responsible for some element of the care transitions process (from either an inpatient, community or primary care perspective). We want to know about the ways in which discharge from these services could be improved. In other words, how to avoid things such as:

- Patients being discharged inappropriately;
- Delayed discharge;
- Not having the correct information shared between services and with patients;
- Patients not having the best support during discharge;

The aim of this interview is to get your views about patient discharge from inpatient mental health services: what procedures would you like to see improved, how discharge can be safer and what role (if any) would you have in improving discharge?

**Ground rules**

- You are being digitally recorded, so speak clearly and do not speak over one another;
- Please respect each others’ right to express a view, even if it differs from yours;
- We will anonymise the transcript so that nobody can be identified by name. However, please try to avoid naming specific healthcare professionals or locations;
- Everything discussed here is confidential.

**Questions**

1. What is important to consider for safely discharging patients from mental health services?
2. Do things go wrong with discharge from inpatient mental health services?

*Follow-up questions: Can you give me any examples of times when discharge has gone wrong? Are there any similarities or differences between the instances where it goes wrong?*

1. Do you think things could go wrong?
2. Do you have a role in helping to prevent things going wrong with discharge?

If yes, probe what role it is. If no, probe why they believe they have a role

*Follow-up questions: Could anyone give an example of a time where you have prevented something going wrong with discharge? How easy/difficult is it for you to do this? Are you prepared to do this? What do you do? Do you feel you have the necessary support? Are any colleagues involved in this as well as / instead of you?*

1. What do you think about having an Expected Discharge Date (EDD) from admission?
2. What do you think about having Clinical Criteria for Discharge (CCD) set at admission?

*Follow-up questions: what do you think about setting these things by assuming ideal recovery? Will it reduce unnecessary waiting? Is unnecessary waiting a concern for patients and clinicians? Who would set the clinical criteria? Would criteria led discharge allow nurses and junior doctors to process discharges? What would the effect of this be? Would this be implementable on your ward? What problems do you for-see with implementing this approach?*

1. To what extent do you think it is important that patients move from any assessment unit (i.e. Crisis, 136 ward) to inpatient ward as early in the day as possible?

*Follow-up questions: Is it important that the first patient should be on the ward by 10am? Is this something that is part of routine care and done automatically? What are the harms (if any) of patients not being discharged before midday? Do you have any experiences where timings of admissions, discharges and transfers have affected patient care? Would this be implementable on your ward? What problems do you for-see with implementing this approach?*

1. What is the ideal time of day to discharge patients?

*Follow-up questions: What (if any) are the benefits for patients being discharged before midday? What are the harms (if any) of patients not being discharged before midday? Is it important that a good proportion of patients are discharged before midday? To what extent do you agree with setting targets of 33% of patients being discharged before midday?*

1. Do you think it’s important to have an MDT meeting for any patient who has been on the ward for longer than 7 days to discuss discharge?

*Follow-up questions: Do you consider 7 days to be an extended length of stay? Do you think it is appropriate for staff to have a home-first mindset in mental health? Would this be implementable on your ward? What problems do you for-see with implementing this approach? What would help increase the likelihood of this being implemented on your ward?*

1. Is it important that each patient is reviewed by a senior clinician who is able to make discharge decisions each day?

*Follow-up questions: Should patients be involved in this daily review? Is it important that this happens before midday? What would happen if this doesn’t happen before midday? Should that person be a psychiatrist each time, or could it be a nurse? Would this be implementable on your ward? What problems do you for-see with implementing this approach? What would help increase the likelihood of this being implemented on your ward?*

1. What are the three most important things healthcare professionals or management/policy makers could do to improve discharge?
2. Are there any other things you would like to do in relation to discharge that might allow people who are ready to be discharged to leave hospital sooner?
3. Are there any other things that healthcare professionals could do to improve safety at discharge?
4. What would help with staff engagement with a new discharge process?
5. How familiar are you with the SAFER patient flow bundle?
6. What do you think would help healthcare professionals engage with a new model of working that is endorsed by NHS England?
7. What are your thoughts on patient written discharge plans?
8. *If not discussed organically during interview*- What has been the effect of the Covid-19 crisis on mental health care transitions?
9. Follow up questions: How has it affected discharge? How has it affected admissions? How has it affected transfers? What practices have changed? How has that affected quality quality and safety
10. Are there any other issues you think we should discuss?

**Topic Guide**

**Patient and Carer Interview**

Background

The aim of this interview is to get your views about discharge from mental health services: what was your experience; what procedures would you like to see improved, how can discharge be safer and what role (if any) would you have in improving discharge?

You or your family/friend have used inpatient mental health services in the past. We want to know how discharge from these services could be improved. In other words, how to avoid things such as:

• Being discharged inappropriately

• Not having the correct information

• Not having the correct support

• Having to wait too long to leave a ward

• Changes to your care plan/treatment without your knowledge or agreement

Ground rules

• As you are being digitally recorded, try to speak clearly and try not to speak over one another;

• Please respect each others’ right to express a view, even if it differs from yours;

• We will anonymise and de-identify the transcript so that nobody can be identified by name. Please try to avoid naming specific healthcare professionals or locations;

• Everything discussed here is confidential.

• Would you like to set any other ground rules?

Questions

1) What is important to consider for safely discharging patients from mental health services?

2) How do things go wrong with discharge from inpatient mental health services?

3) How might things go wrong?

4) Do you have a role in helping to prevent things going wrong with your/your family members discharge?

Follow-up questions: How easy/difficult is it for you to do this? Are you prepared or confident to do this? What might you do?

5) What do you think about having an Expected Discharge Date (EDD) from admission? This is a estimated date that is set at admission by a clinician, it is not definite but a goal for everybody to work towards.

6) What do you think about having Clinical Criteria for Discharge (CCD) set at admission? This is a set of criteria that a patient would meet to be considered ready for discharge.

Follow-up questions: what do you think about setting these criteria, do they assume an ideal recovery? What might be the benefits or risks of setting these early? When might be the best time to set these criteria? Will it reduce unnecessary waiting? Is unnecessary waiting a concern for patients and familys/carers?

7) Is it important that patients move from any assessment unit (i.e. Crisis, 136 ward) to inpatient ward as early in the day as possible?

Follow-up questions: Is it important that the first patient should be on the ward by 10am? Do you have any experiences where timings of admissions, discharges and transfers have affected your care?

8) What is the ideal time of day to be discharged?

Follow-up questions: do you think it is important to be discharged before midday? Is it important that a good proportion of patients are discharged before midday? Do you agree with setting targets of 33% of patients being discharged before midday?

9) On general medicine wards, multi-professional groups of staff (I.e. doctor, nurse, physiotherapist, pharmacist) often meet to discuss patients who have been on a ward with extended stays (in some cases over 7 days). Do you think this practice would also be applicable to a mental health ward?

Follow up: What length of stays do you think would best for a mental health ward discharge meeting? Do you consider 7 days to be an extended length of stay? Do you think it is appropriate for staff to have a focus on getting patients ready for discharge?

10) Is it important that each patient is reviewed by a senior clinician who is able to make discharge decisions each day during their hospital stay?

Follow-up questions: Should patients be involved in this daily review? Is it important that this happens before midday? Should that person by a psychiatrist each time, or could it be a nurse?

11) What three things could healthcare professionals working on the ward do to improve your discharge?

12) Are there any other things you would like healthcare professionals to do in relation to discharge that might allow people who are ready to be discharged to leave hospital sooner?

13) Are there any other things you would like healthcare professionals to do to improve safety at discharge?

14) *If not discussed organically during interview*- What has been the effect of the Covid-19 crisis on mental health care transitions?

Follow up questions: How has it affected discharge? How has it affected admissions? How has it affected transfers? What practices have changed? How has that affected quality quality and safety

15) Are there any other issues you think we should discuss?

**Safer Mental Health Discharge: Stakeholder Interviews and Consensus Study**

**Topic Guide**

**Key Informants Interview**

Background

You are a group of professionals working in relation to mental health services. You do not deliver frontline care to patients, but are involved in decisions, policy and implementation of mental health services. We want to know ways in which discharge from these services could be improved. In other words, how to avoid things such as:

• Patients being discharged inappropriately;

• Delayed discharge;

• Not having the correct information shared between services and with patients;

• Not having the correct support;

The aim of this interview is to get your views about discharge from inpatient mental health services: what procedures would you like to see improved, how discharge can be safer and what role (if any) would you have in improving discharge?

Ground rules

• You are being digitally recorded, so speak clearly and do not speak over one another;

• Please respect each others’ right to express a view, even if it differs from yours;

• We will anonymise the transcript so that nobody can be identified by name. However, please try to avoid naming specific healthcare professionals or locations;

• Everything discussed here is confidential.

Questions

1) What is important to consider for safely discharging patients from mental health services?

2) Do you know of any things that can wrong with discharge from inpatient mental health services?

3) Do you think things could go wrong?

4) Do you have a role in helping to prevent things going wrong with discharge?

Follow-up questions: How easy/difficult is it for you to do this? Are you prepared to do this? What do you do?

5) What do you think about having an Expected Discharge Date (EDD) from admission?

6) What do you think about having Clinical Criteria for Discharge (CCD) set at admission?

Follow-up questions: What do you think about setting these things by assuming ideal recovery? Will it reduce unnecessary waiting? Is unnecessary waiting a concern for patients and clinicians? Who would set the clinical criteria? Would criteria led discharge allow nurses and junior doctors to process discharges? What would the effect of this be? Would this be implementable in your organisation (or affiliated services)? What problems do you for-see with implementing this approach?

7) Do you think it is important that patients move from any assessment unit (i.e. Crisis, 136 ward) to inpatient ward as early in the day as possible?

Follow-up questions: Is it important that the first patient should be on the ward by 10am? Do you have any experiences where timings of admissions, discharges and transfers have affected patient care? Would this be implementable on your organisation (or affiliated services)? What problems do you for-see with implementing this approach?

8) What is the ideal time of day to discharge patients?

Follow-up questions: What (if any) are the benefits for patients being discharged before midday? Is it important that a good proportion of patients are discharged before midday? Do you agree with setting targets of 33% of patients being discharged before midday?

9) Do you think it’s important to have an MDT meeting for any patient who has been on the ward for longer than 7 days to discuss discharge?

Follow-up questions: Do you consider 7 days to be an extended length of stay? Do you think it is appropriate for staff to have a home-first mindset in mental health? Would this be implementable on your ward? What problems do you for-see with implementing this approach?

10) Is it important that each patient is reviewed by a senior clinician who is able to make discharge decisions each day?

Follow-up questions: Should patients be involved in this daily review? Is it important that this happens before midday? Should that person by a psychiatrist each time, or could it be a nurse? Would this be implementable on your ward? What problems do you for-see with implementing this approach?

11) What three things could healthcare professionals or management/policy makers do to improve discharge?

12) Are there any other things you would like to do in relation to discharge that might allow people who are ready to be discharged to leave hospital sooner?

13) Are there any other things you would like healthcare professionals to do to improve safety at discharge?

14) What would help with staff engagement with a new discharge process?

15) Are you familiar with the SAFER patient flow bundle?

16) What are your thoughts on patient written discharge plans?

17) *If not discussed organically during interview*- What has been the effect of the Covid-19 crisis on mental health care transitions?

Follow up questions: How has it affected discharge? How has it affected admissions? How has it affected transfers? What practices have changed? How has that affected quality quality and safety

18) Are there any other issues you think we should discuss?
