# Supplementary material for: Effects of the first COVID-19 lockdown on quality and safety in mental healthcare transitions in England
Source: BJPsych Open. 2021 Aug 31;7(5):e156. doi: 10.1192/bjo.2021.996 (PMC8410739; doi:10.1192/bjo.2021.996)
Supplement: Supplementary file 1 [file bjosup.zip › S2056472421009960sup003.docx]

**Participant Sociodemographic Details**

| **Participant ID** | **Year of birth** | **Age** | **Gender** | **Ethnicity** | **Group1** | **Group 2** | **Job Title and Experience** |
| --- | --- | --- | --- | --- | --- | --- | --- |
| **2** | **1966** | **54** | **F** | **White British** | **HCP** | **NR** | **Ward Manager 36 years** |
| **1** | **1961** | **59** | **F** | **White British** | **Carer** | **NR** | **NR** |
| **6** | **1957** | **63** | **F** | **White British** | **HCP** | **NR** | **Staff nurse** |
| **4** | **1985** | **35** | **M** | **Black African** | **KI** | **NR** | **Mental Health Lawyer 10 years** |
| **15** | **1966** | **54** | **F** | **White British** | **HCP** | **NR** | **Ward Manager 20 years** |
| **16** | **1995** | **25** | **M** | **White British** | **SU** | **NR** | **NR** |
| **17** | **1974** | **46** | **M** | **Black Carribean** | **Carer** | **Advocate** | **NR** |
| **18** | **1982** | **38** | **M** | **White British** | **SU** | **NR** | **NR** |
| **19** | **1968** | **52** | **F** | **White British** | **HCP** | **SU and KI (service manager)** | **Consultant clinical psychologist and service manager** |
| **20** | **1979** | **41** | **F** | **White Irish** | **KI** | **HCP** | **Secretary for MH interface prescriving forum (MH pharmacist and CCG)** |
| **21** | **1971** | **49** | **F** | **White British** | **Carer** | **NR** |  |
| **Gavin 6** | **1974** | **46** | **F** | **White British** | **SU** | **Carer** |  |
| **Gavin 8** | **1989** | **31** | **M** | **White British** | **KI** |  | **Policy professional 4 years** |
| **Gavin 7** | **1955** | **65** | **F** | **White British** | **KI** |  | **Policy advisor, 37 years’ experience of mental health information and policy work** |
| **Gavin 1** | **1986** | **34** | **F** | **White British** | **HCP** | **NR** | **Ward Manager 10 years** |
| **Gavin 2** | **1982** | **38** | **F** | **White British** | **Carer** | **Patient** |  |
| **Gavin 3** | **1974** | **46** | **F** | **Black African** | **HCP** | **NR** | **HCA 10 years** |
| **Gavin 4** | **1965** | **55** | **F** | **White British** | **Carer** | **NR** |  |
| **Gavin 5** | **1983** | **37** | **F** | **White British** | **HCP** |  | **MH nurse 12 years** |
| **22** | **1978** | **42** | **F** | **White British** | **KI** | **HCP** | **MH transitions researcher and MH nurse/lecturer 20 years** |
| **23** | **1982** | **38** | **M** | **Middle Eastern** | **HCP** | **SU** | **Psychiatrist** |
| **24** | **1965** | **55** | **F** | **White British** | **HCP** | **NR** | **Lead pharmacist 27 years** |
| **25** | **1978** | **42** | **M** | **White British** | **Carer** |  |  |
| **26** | **1968** | **52** | **F** | **White Irish** | **SU** | **KI (Advocate)** | |
| **27** | **1954** | **66** | **M** | **White British** | **HCP** | **KI** | **Pharmacy Advisor (previous lead pharmacist)** |
| **28** | **1958** | **62** | **F** | **White British** | **KI** | **Carer** | **CQC inspector (lived experience)** |
| **29** | **1982** | **38** | **F** | **White British** | **HCP** | **Carer** | **OT- 11 years** |
| **30** | **1963** | **57** | **F** | **White Other** | **HCP** |  | **Lead pharmacist 7 years in MH** |
| **31** | **1991** | **29** | **M** | **White British** | **SU** | **NR** |  |
| **32** | **1974** | **46** | **F** | **White Other** | **Carer** |  |  |
| **33** | **1982** | **38** | **F** | **White British** | **KI** | **HCP** | **Head of nursing 10 years** |
| **34** | **1977** | **43** | **F** | **White British** | **HCP** |  | **Consultant Liaison Psychiatrist 7 years** |
| **35** | **1977** | **43** | **M** | **White British** | **SU** | **KI** | **Researcher MH (5 years)** |
| **36** | **1980** | **40** | **M** | **White British (Scottish)** | **HCP** |  | **Consultant Clinical psychologist 17 years** |

| **Participant ID** | **Age** | **Gender** | **Ethnicity** | **Primary Group** | **Secondary Group (if applicable)** | **Job Title and Years Experience (if disclosed)** |
| --- | --- | --- | --- | --- | --- | --- |
| 1 | 59 | F | White British | Carer | NR | NR |
| 2 | 54 | F | White British | HCP | NR | Ward Manager 36 years |
| 4 | 35 | M | Black African | KI | NR | Mental Health Lawyer 10 years |
| 6 | 63 | F | White British | HCP | NR | Staff nurse |
| 15 | 54 | F | White British | HCP | NR | Ward Manager 20 years |
| 16 | 25 | M | White British | SU | NR | NR |
| 17 | 46 | M | Black Carribean | Carer | KI (Advocate) | NR |
| 18 | 38 | M | White British | SU | NR | NR |
| 19 | 52 | F | White British | HCP | SU and KI (service manager) | Consultant clinical psychologist and service manager |
| 20 | 41 | F | White Irish | KI | HCP | Secretary for MH interface prescriving forum (MH pharmacist and CCG) |
| 21 | 49 | F | White British | Carer | NR |  |
| 22 | 42 | F | White British | KI | HCP | MH transitions researcher and MH nurse/lecturer 20 years |
| 23 | 38 | M | Middle Eastern | HCP | SU | Psychiatrist |
| 24 | 55 | F | White British | HCP | NR | Lead pharmacist 27 years |
| 25 | 42 | M | White British | Carer |  |  |
| 26 | 52 | F | White Irish | SU | KI (Advocate) |  |
| 27 | 66 | M | White British | HCP | KI | Pharmacy Advisor (previous lead pharmacist) |
| 28 | 62 | F | White British | KI | Carer | CQC inspector (lived experience) |
| 29 | 38 | F | White British | HCP | Carer | OT- 11 years |
| 30 | 57 | F | White Other | HCP |  | Lead pharmacist 7 years in MH |
| 31 | 29 | M | White British | SU | NR |  |
| 32 | 46 | F | White Other | Carer |  |  |
| 33 | 38 | F | White British | KI | HCP | Head of nursing 10 years |
| 34 | 43 | F | White British | HCP |  | Consultant Liaison Psychiatrist 7 years |
| 35 | 43 | M | White British | SU | KI | Researcher MH (5 years) |
| 36 | 40 | M | White British (Scottish) | HCP |  | Consultant Clinical psychologist 17 years |
| G1 | 34 | F | White British | HCP | NR | Ward Manager 10 years |
| G2 | 38 | F | White British | Carer | SU |  |
| G3 | 46 | F | Black African | HCP | NR | HCA 10 years |
| G4 | 55 | F | White British | Carer | NR |  |
| G5 | 37 | F | White British | HCP |  | MH nurse 12 years |
| G6 | 46 | F | White British | SU | Carer |  |
| G7 | 65 | F | White British | KI |  | Policy advisor, 37 years’ experience of mental health information and policy work |
| G8 | 31 | M | White British | KI |  | Policy professional 4 years |

Key

HCP- Healthcare Professional

SU- Service User

KI- Key informant

NR- Not reported

* Participants with IDs G1-8 were interviewed by GDW, all other participants were interviewed by NT, these interviews were conducted during a period of leave after (1-2) which is why the numbers are not aligned.
